# Association between periodontal disease and systemic diseases: a cross-sectional analysis of current evidence

**DOI:** 10.1186/s40779-024-00583-y

**Published:** 2024-12-04

**Authors:** Di Huang, Yun-Yun Wang, Bing-Hui Li, Lan Wu, Wen-Zhong Xie, Xia Zhou, Bin Ma

**Affiliations:** 1 Evidence-Based Medicine Center, School of Basic Medical Sciences, Lanzhou University, Lanzhou, 730000 China; 2 Center for Evidence-Based and Translational Medicine, Zhongnan Hospital of Wuhan University, Wuhan, 430071 China; 3 Department of Stomatology, Zhongnan Hospital of Wuhan University, Wuhan, 430071 China; 4 Henan Provincial Engineering Research Center for Microecological Regulatory of Oral Environment and Oral Implantology, Kaifeng University Health Science Center, Kaifeng, 475000 Henan China; 5 Department of Stomatology, Daping Hospital, Army Medical University, Chongqing, 400042 China; 6 Research Center for Medical Device Regulatory Science, Lanzhou University, Lanzhou, 730000 China

**Keywords:** Periodontal disease, Periodontitis, Cancer, Cardiovascular diseases, Metabolic disorders, Neurological conditions, Evidence analysis

## Abstract

**Background:**

Numerous systematic reviews and meta-analyses have been published that evaluate the association between periodontal disease and systemic diseases, many of which address similar topics. Moreover, their quality requires assessment. Therefore, we performed a cross-sectional analysis to examine the evidence on the relationship between periodontal disease and systemic diseases.

**Methods:**

The PubMed, Embase, Web of Science, and the Cochrane Library databases were systematically searched to identify relevant systematic reviews and meta-analyses. Only studies that considered periodontal disease as the exposure factor and various systemic diseases as the outcome were included. The basic characteristics and pertinent data from the selected studies were extracted. The modified version of A Measurement Tool to Assess Systematic Reviews 2 (AMSTAR 2) was employed for quality assessment, while R software was used for statistical analysis.

**Results:**

Among the 212 relevant systematic reviews and meta-analyses, 57 were finally included in our analysis. These studies involved 75 diseases and 81 disease-related outcomes, with cancer (19/81) being the most frequently addressed topic. Of the 81 outcomes, 67 demonstrated a significant association. Notably, the highest risk estimate was found for head and neck cancer [odds ratio (*OR*) = 3.17, 95% confidence interval (CI) 1.78 − 5.64], while the lowest was observed for premature rupture of the amniotic sac [relative risk (*RR*) = 1.10, 95% CI 1.08 − 1.12]. The methodological quality ratings indicated that approximately 71.93% of included studies were classified as “Critically low”, with another 17.54% rated as “Low”, and only about 10.53% categorized as “Moderate”.

**Conclusions:**

Periodontal disease significantly elevates the risks associated with 15 cancer-related, 8 cardiovascular-related, 8 metabolic-related, and 5 neurological-related outcomes. However, the overall methodological quality of existing systematic reviews and meta-analyses is generally suboptimal and requires enhancement to generate higher-quality evidence in the future.

**Supplementary Information:**

The online version contains supplementary material available at 10.1186/s40779-024-00583-y.

## Background

Periodontal disease is a prevalent oral disease characterized by gingival inflammation and the destruction of periodontal tissues. It encompasses a spectrum of disorders, ranging from gingivitis, which is confined to gum inflammation, to periodontitis, which involves deeper tissue destruction and can be classified into mild to moderate and moderate to advanced stages. These conditions may ultimately lead to both tooth loss and an increased burden of disease [1, 2]. An increasing number of studies have shown that periodontal disease is associated with multiple systemic diseases, including cardiovascular disease, diabetes mellitus, hypertension, respiratory disorders, preterm birth, and low birth weight [3–7].

Although periodontal disease primarily affects the oral cavity, substantial evidence suggests that it may impact systemic health through mechanisms involving innate and adaptive immune responses [8, 9]. In the early stages of gingivitis, oral microbial dysbiosis can promote persistent inflammation. Local inflammatory responses facilitate the entry of pathogens, such as *Porphyromonas gingivalis*, into the bloodstream, potentially affecting adjacent tissues. As periodontal disease progresses, the host’s immune response intensifies, influencing systemic immune pathways and potentially leading to autoimmune disorders and other systemic diseases. Currently, the mechanisms by which periodontal disease influences systemic conditions remain unclear. The two prevailing theories propose that periodontal disease induces a systemic inflammatory response or that periodontal pathogens or their metabolites spread through the bloodstream to various parts of the body directly [8, 9].

Numerous systematic reviews have explored the association between periodontal disease and various other diseases. Despite addressing similar topics, the conclusions remain controversial due to differences in study design, population characteristics, and diagnostic criteria for diseases [10, 11]. Additionally, the methodological quality of these systematic reviews is often unclear and requires thorough evaluation. To enhance the utilization of evidence, it is necessary to synthesize these findings. This study aims to analyze the existing systematic reviews and meta-analyses regarding the relationship between periodontal disease and systemic diseases, explore the impact of periodontal disease on systemic diseases, comprehensively assess their quality, and evaluate as well as summarize the strength of this correlation.

## Methods

### Inclusion and exclusion criteria

Inclusion criteria comprising: 1) the research subjects must be human participants; 2) the exposure factor is periodontal disease; 3) the outcome must be a specific systemic disease, such as cancer, coronary heart disease, diabetes mellitus, or stroke; 4) the study design of interest involves non-interventional systematic review or meta-analysis. Only studies published in Chinese or English with accessible full text were included.

Studies presented solely in abstract form, encompassing meeting reports or protocols were excluded. For studies addressing the same topic, both the number of original studies included and the publication date were comprehensively considered during screening. Generally, preference was given to the most recent meta-analysis that contained the largest number of original studies.

### Search strategy

This study conducted a comprehensive evaluation of systematic reviews and meta-analyses regarding the association between periodontal disease and systemic diseases. The PubMed, Embase, Web of Science, and the Cochrane Library databases were independently searched until May 31, 2024. The search strategy incorporated Medical Subject Headings (MeSH) as well as free text terms (title/abstract) related to: 1) periodontal disease, such as “periodontal disease”, “periodontitis”, “gingivitis”, “periodontal attachment loss”, “alveolar bone loss”, “clinical attachment loss”; and 2) study design, including “systematic review” and “meta-analysis”. No language restrictions were initially imposed. A complementary screening of the references from the analyzed studies was also performed to include any additional relevant studies.

### Data extraction and quality assessment

Three researchers collaboratively retrieved the literature based on predefined inclusion and exclusion criteria. The following information was extracted: first author, year of publication, journal name, study population, number of included studies for each systematic review, ascertainment of exposure and outcomes, whether a meta-analysis was conducted or not, and relevant data on outcomes. Disagreements were resolved through consultation with a third party or discussion among the researchers.

The methodological quality of included systematic reviews and meta-analyses was assessed using a modified version of A Measurement Tool to Assess Systematic Reviews 2 (AMSTAR 2, Additional file 1: Table S1), which comprises 16 evaluation items (Q1 to Q16) that assess the risk of bias and heterogeneity in the included studies [12]. The overall quality of analyzed studies was recorded from high to low as “High”, “Moderate”, “Low”, and “Critically low”. Two researchers cooperatively conducted these quality assessments. Disagreements were addressed through discussion, and if consensus could not be reached, a third-party expert was consulted for a final decision.

### Statistical analysis

Each study was reviewed in detail, focusing on the design, methods, and results to analyze the association between periodontal disease and various systemic diseases. A Microsoft Excel spreadsheet was utilized to compile the basic information of included studies. Based on this data, we performed a comprehensive analysis that included examining the publication year and source journal of the studies, counting the number of original studies included in the individual systematic review, describing the ascertainment of exposure and outcomes, as well as assessing the use of meta-analysis. Categorical items were presented as frequencies and percentages. A bar chart was generated to illustrate the results of methodological quality evaluation. All systemic diseases were classified according to the International Classification of Diseases 11th Revision (ICD-11). Tableau was employed for visualizing disease classification, while R software (version 4.3.2) along with the “forestploter” package (version 1.1.2) was used for evidence analysis and generating forest plots.

## Results

### Basic characteristics

**Fig. 1:**
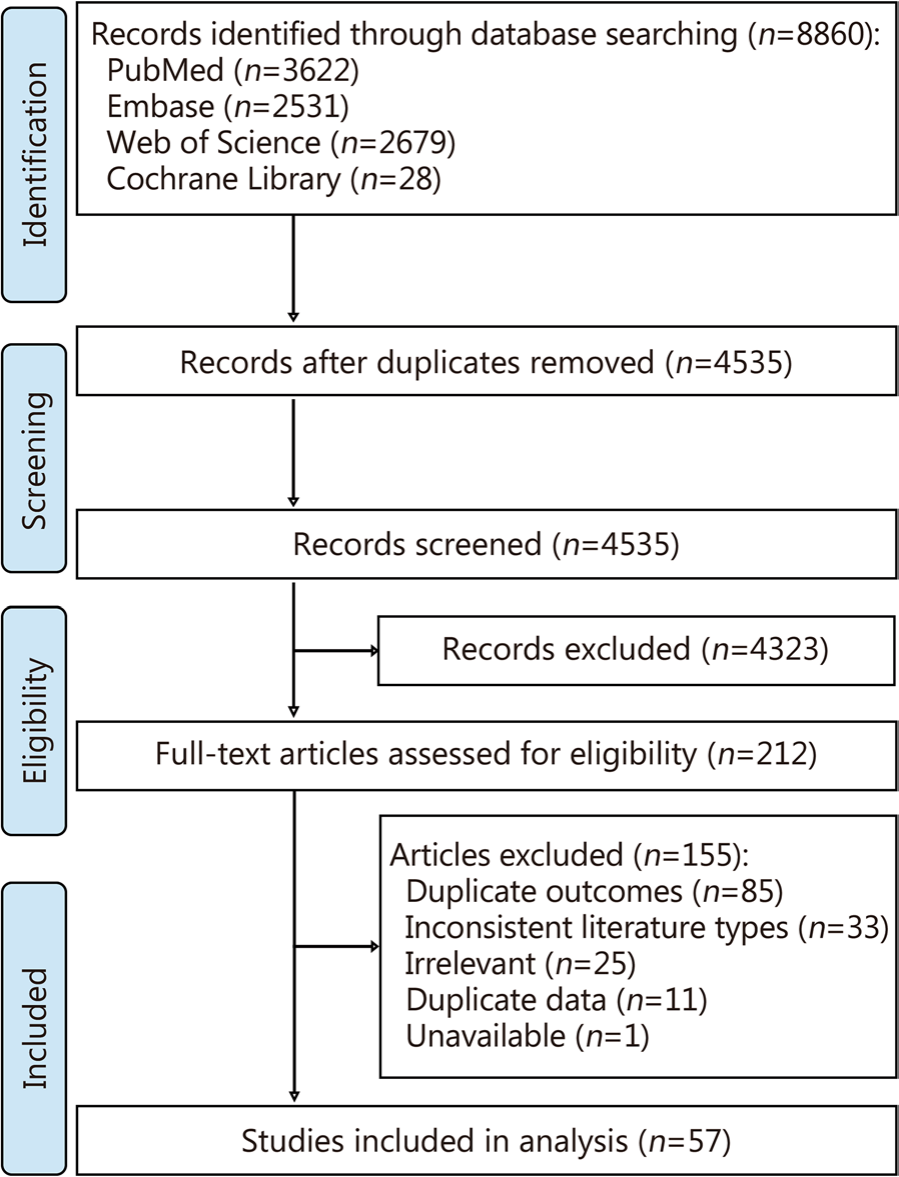
Flow diagram of study selection

**Fig. 2:**
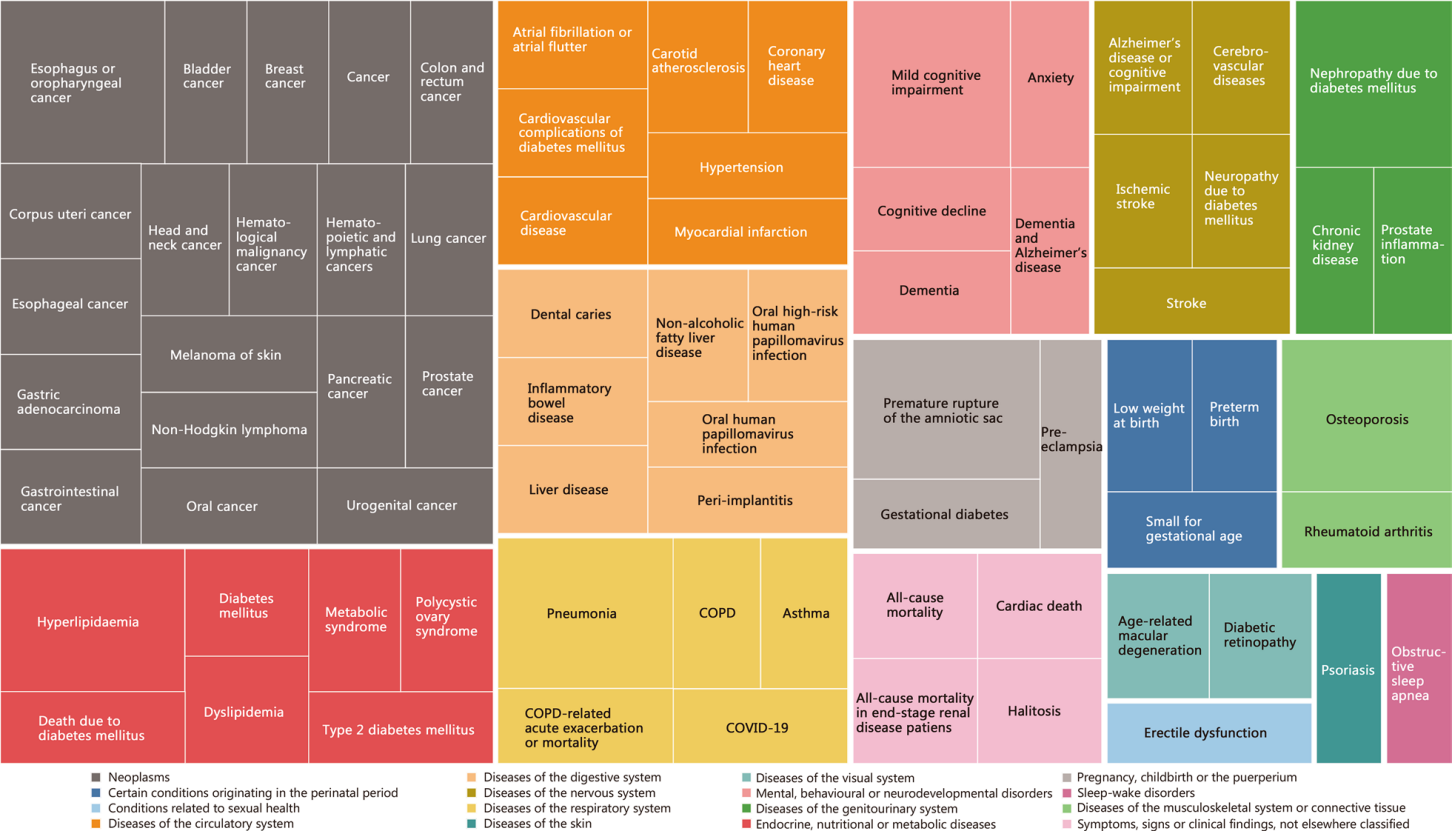
Treemap of disease distribution. COPD chronic obstructive pulmonary disease, COVID-19 coronavirus disease 2019

Based on the predefined inclusion and exclusion criteria, a total of 57 systematic reviews and meta-analyses examining the relationship between periodontal disease and systemic diseases were finally included [13–69] (Fig. 1). The basic characteristics of these studies were presented in Additional file 1: Table S2. Among the 57 systematic reviews, the majority (87.72%) were published after 2020, with 40 (70.18%) including more than 10 original studies, 51 (89.47%) reporting the ascertainment of exposure and outcomes, and 54 (94.74%) conducting a meta-analysis for data synthesis. These studies appeared in a total of 48 journals, primarily within the fields of oral medicine and general medicine (Table 1). A cumulative total of 75 diseases were reported and classified into 16 categories, with neoplasms being the most common category (*n* = 19), followed by circulatory system diseases (*n* = 7), digestive system diseases (*n* = 7), and endocrine, nutritional or metabolic diseases (*n* = 7) (Fig. 2).

**Table 1:** Basic characteristics of included systematic reviews (*n* = 57)

Item	*n* (%)
Publication year of included systematic reviews	
2024	8 (14.04)
2023	10 (17.54)
2022	7 (12.28)
2021	9 (15.79)
2020	16 (28.07)
2016 − 2019	7 (12.28)
Journals where included systematic reviews were published*	
Oral Dis	3 (5.26)
Acta Odontol Scand	2 (3.51)
BMC Oral Health	2 (3.51)
Clin Oral Investig	2 (3.51)
J Clin Med	2 (3.51)
J Clin Periodontol	2 (3.51)
Med Oral Patol Oral Cir Bucal	2 (3.51)
PLoS One	2 (3.51)
Number of included studies for individual systematic review	
> 50	6 (10.53)
40 − 50	1 (1.75)
30 − 39	5 (8.77)
20 − 29	6 (10.53)
10 − 19	22 (38.60)
≤ 9	17 (29.82)
Ascertainment of exposure and outcome	
Yes	51 (89.47)
No	6 (10.53)
Use of meta-analysis for individual systematic review	
Yes	54 (94.74)
No	3 (5.26)

*Only journals that published more than 2 systematic reviews were shown in the table. The full list of journals can be seen in Additional file 1: Table S2

### Methodological quality

**Fig. 3:**
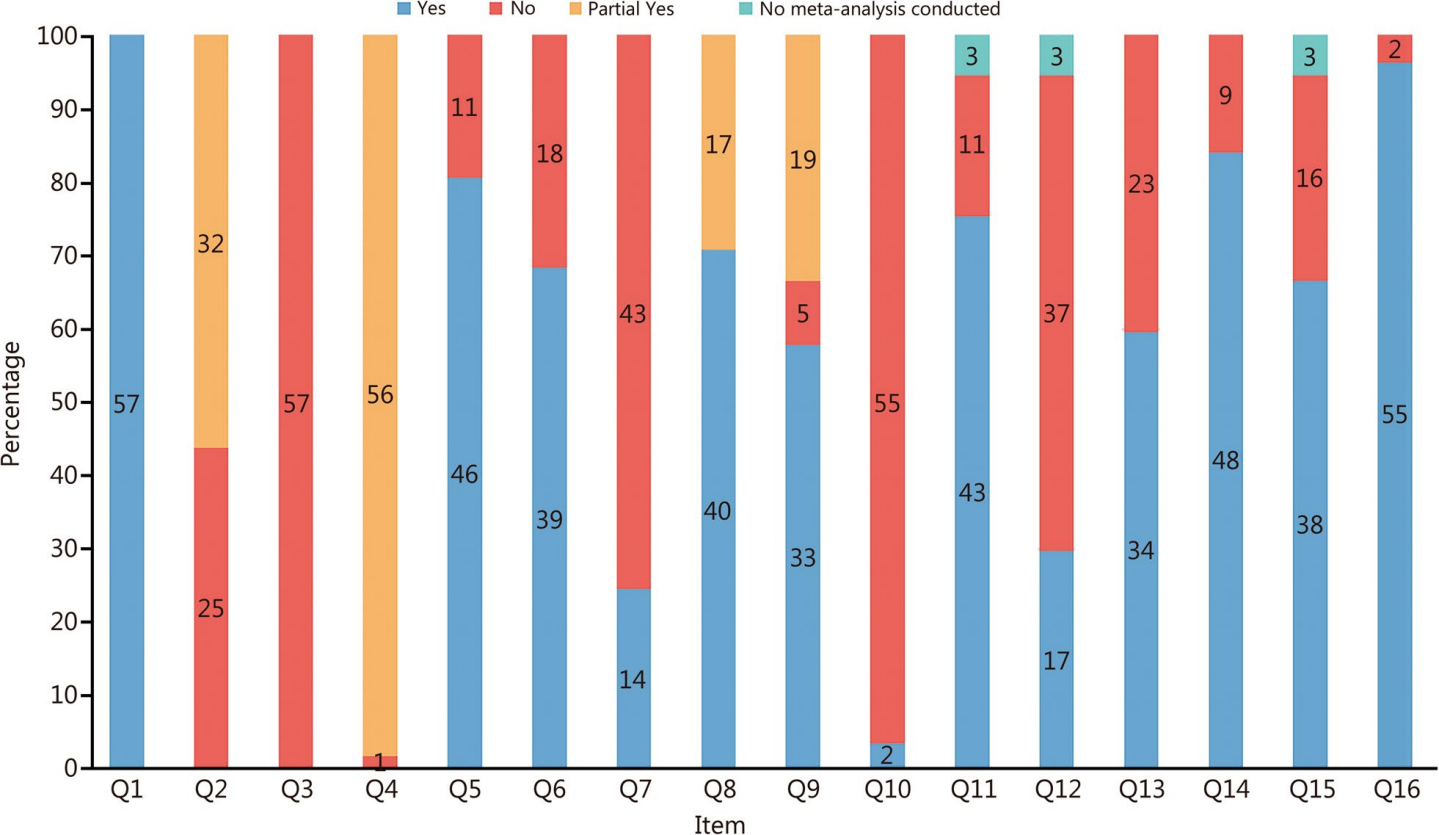
Distribution of the results across the 16 evaluation items. Q1 − Q16 16 evaluation items of the modified version of A Measurement Tool to Assess Systematic Reviews 2

This study utilized a modified version of the AMSTAR 2 tool to evaluate the methodological quality of the included studies. The distribution of results across the 16 evaluation items is shown in Fig. 3. The findings revealed that all studies exhibited varying degrees of methodological flaws (Additional file 1: Table S3). Overall, the methodological quality was rated as “Critically low” (71.93%), “Low” (17.54%), and “Moderate” (10.53%). Notably, nearly all studies demonstrated deficiencies in critical areas, including “explain their selection of the study designs for inclusion” (Q3) and “report on the sources of funding for the studies included” (Q10). The majority only partially satisfied or failed to meet the criteria, particularly in key areas such as “provide a list of excluded studies and justify the exclusions” (Q7) (only 24.56% provided a list of excluded studies with justifications) and “assess the potential impact of risk of bias in individual studies on the results of the meta-analysis or other evidence synthesis” (Q12) (only 31.48% of the 54 studies that underwent meta-analysis assessed potential bias impacts). These findings suggest that the methodological quality of current systematic reviews on periodontal disease and various systemic diseases is generally inadequate. Therefore, future research should address these deficiencies to enhance evidence reliability.

### Evidence analysis

**Fig. 4:**
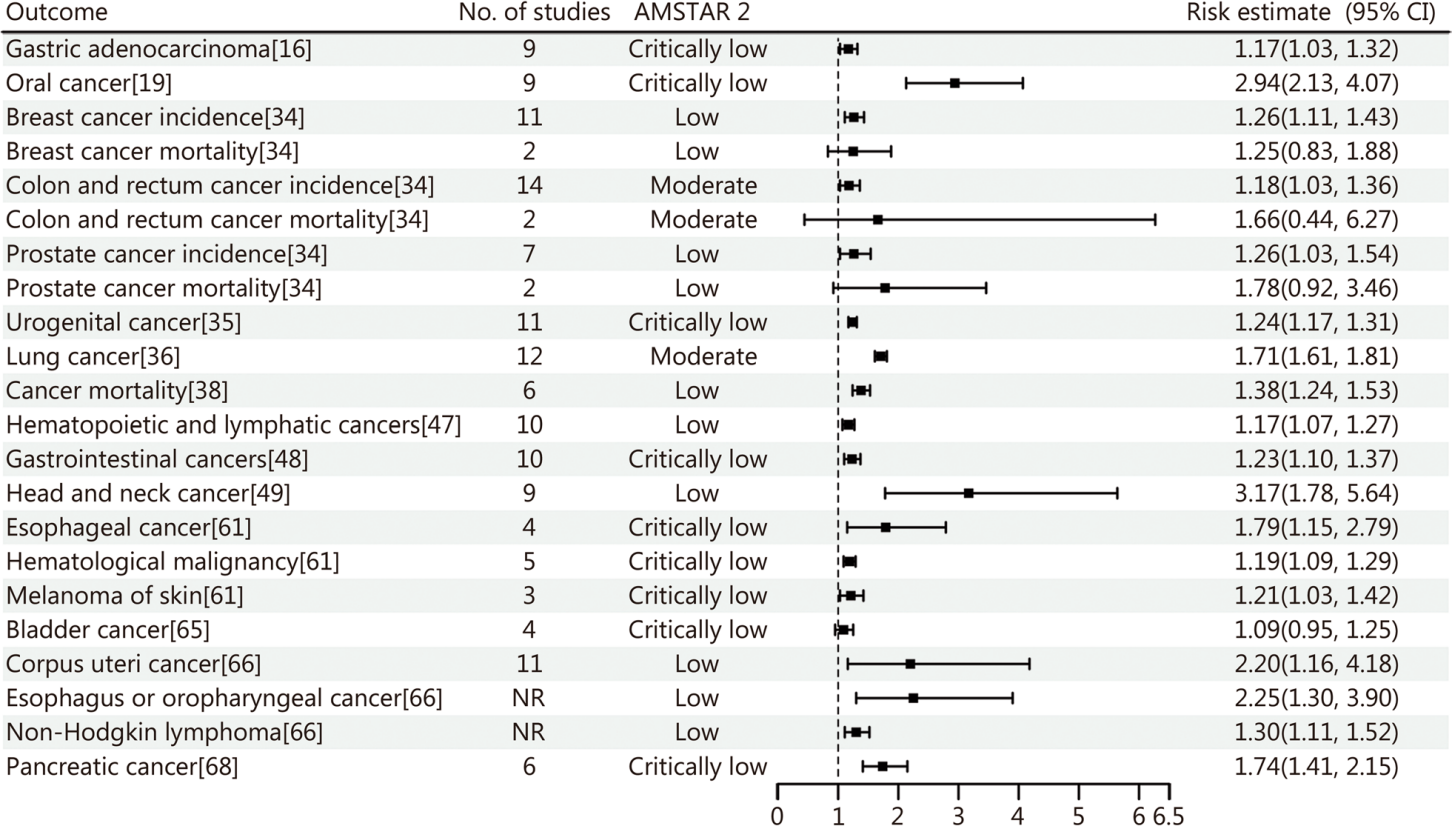
Forest plot of the association between periodontal disease and cancer. No. number, NR no report, AMSTAR 2 A Measurement Tool to Assess Systematic Reviews 2, CI confidence interval

The findings of this study regarding the association between periodontal disease and systemic diseases indicate that periodontal disease significantly elevates the risk of various conditions (Additional file 1: Table S2). Specifically, periodontal disease is notably linked to an increased risk of several cancers, including head and neck cancer [odds ratio (*OR*) = 3.17, 95% CI 1.78 − 5.64], oral cancer (*OR* = 2.94, 95% CI 2.13 − 4.07), and esophagus or oropharyngeal cancer [hazard ratio (*HR*) = 2.25, 95% CI 1.30 − 3.90]. Although the association between periodontal disease and mortality from certain cancers (breast, prostate, colon and rectum) was not statistically significant due to high levels of heterogeneity, the overall results still suggest a link between periodontal disease and cancer risk (Fig. 4).

**Fig. 5:**
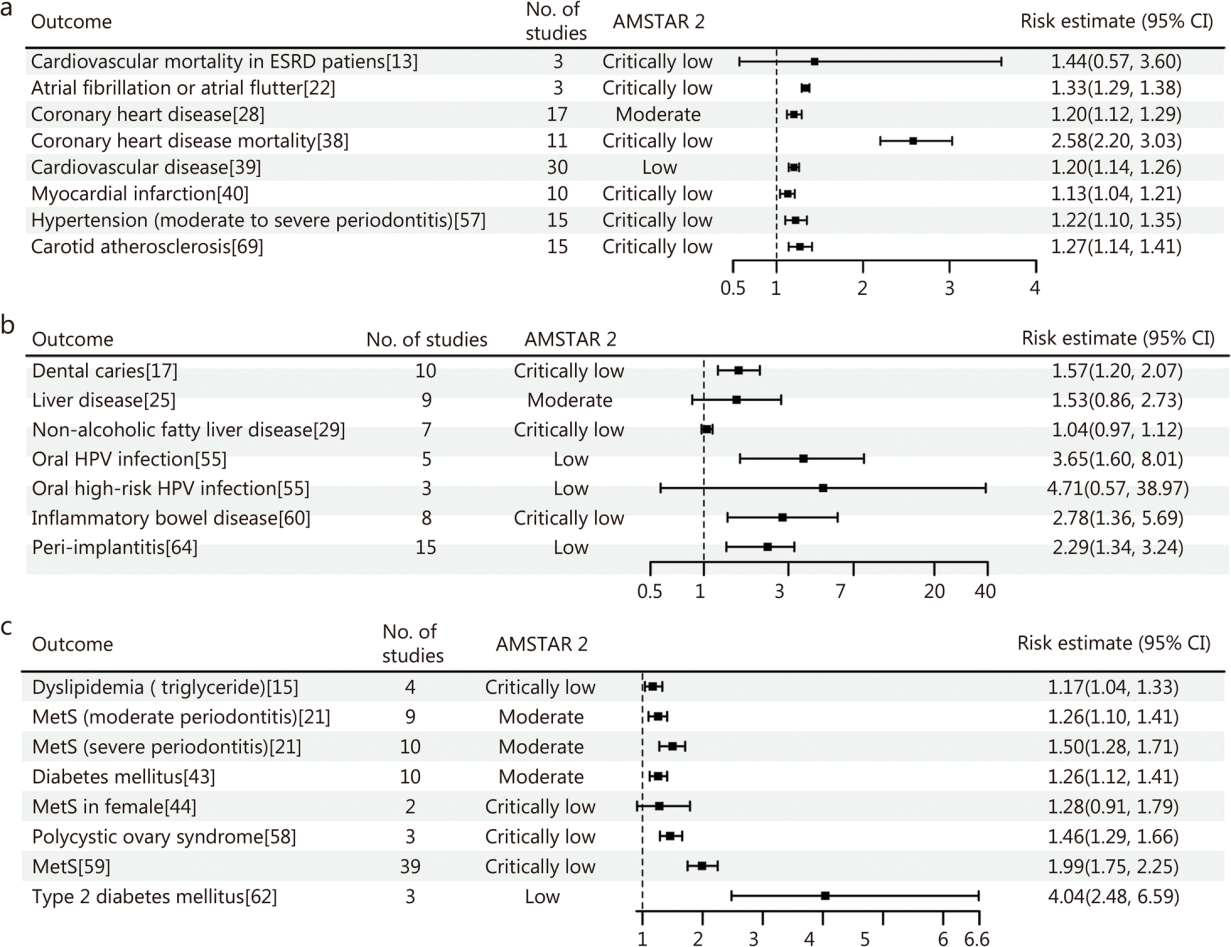
Forest plot of the association between periodontal disease and circulatory system diseases (**a**), digestive system diseases (**b**), and metabolic diseases (**c**). ESRD end-stage renal disease, HPV human papillomavirus, MetS metabolic syndrome, No. number, AMSTAR 2 A Measurement Tool to Assess Systematic Reviews 2, CI confidence interval

Periodontal disease may increase the risk of cardiovascular disease, including coronary heart disease [relative risk (*RR*) = 1.20, 95% CI 1.12 − 1.29], myocardial infarction (*RR* = 1.13, 95% CI 1.04 − 1.21), atrial fibrillation or atrial flutter (*RR* = 1.33, 95% CI 1.29 − 1.38), carotid atherosclerosis (*OR* = 1.27, 95% CI 1.14 − 1.41), and hypertension (*OR* = 1.22, 95% CI 1.10 − 1.35). However, limited evidence indicates a nonsignificant association between periodontal disease and cardiovascular mortality in patients with end-stage renal disease (ESRD; *HR* = 1.44, 95% CI 0.57 − 3.60) (Fig. 5a). Regarding digestive system diseases, periodontal disease significantly elevates the risk of inflammatory bowel disease (*RR* = 2.78, 95% CI 1.36 − 5.69), dental caries (*OR* = 1.57, 95% CI 1.20 − 2.07) and peri-implantitis (*OR* = 2.29, 95% CI 1.34 − 3.24). However, no relationship was observed between periodontal disease and liver disease. Current evidence demonstrates that periodontal disease increases the risk of oral human papillomavirus (HPV) infection by 3.65 times, but the link between periodontal disease and high-risk oral HPV infections remains inconclusive (Fig. 5b). In terms of metabolic diseases, periodontal disease notably raises the risk of metabolic syndrome, particularly in patients with moderate to severe periodontitis. Nevertheless, in certain female populations, the association between periodontal disease and metabolic syndrome did not achieve statistical significance (Fig. 5c).

**Fig. 6:**
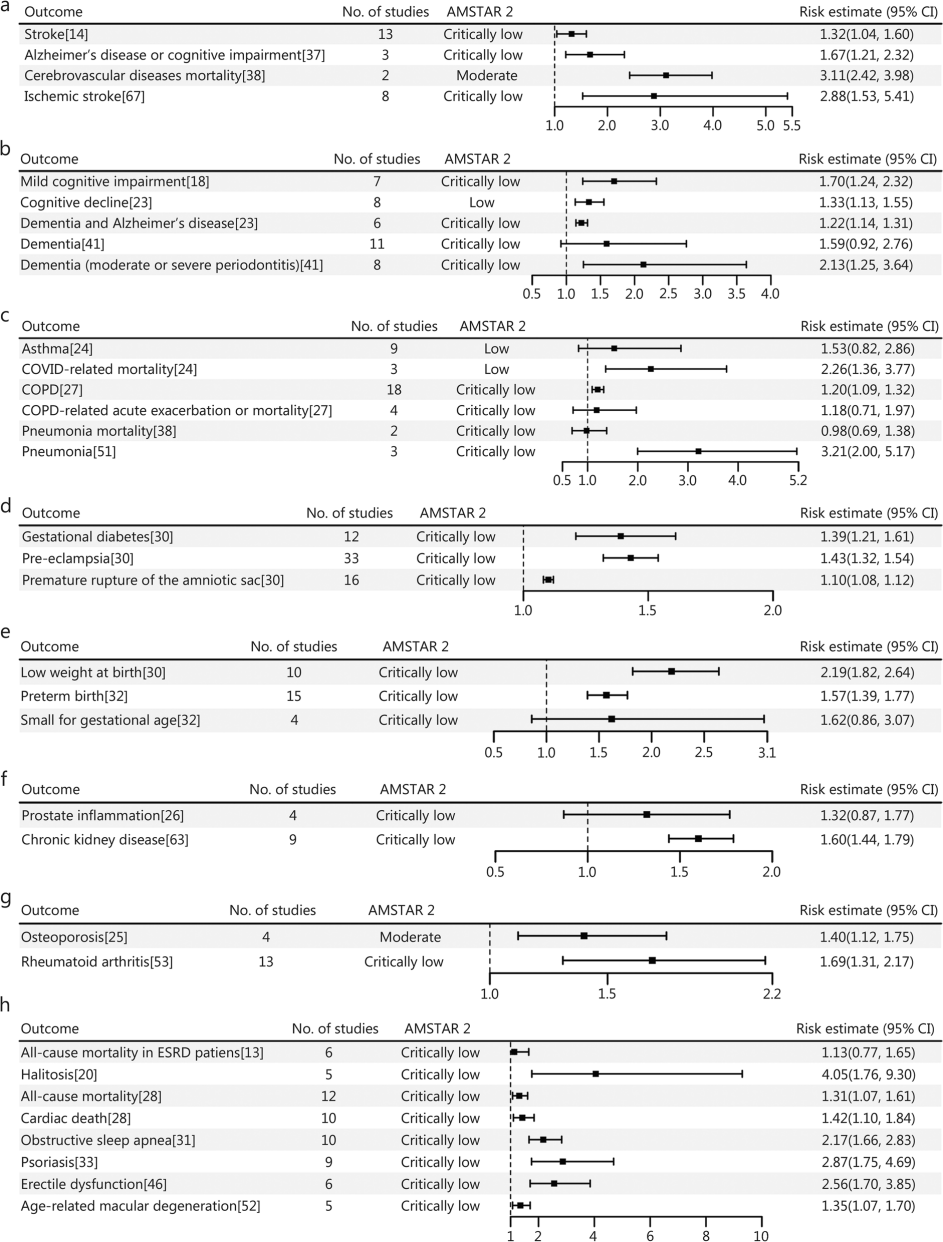
Forest plot of the association between periodontal disease and multiple diseases. **a** nervous system diseases. **b** mental, behavioral or neurodevelopmental disorders. **c** respiratory system diseases. **d** pregnancy, childbirth, or the puerperium diseases. **e** perinatal period diseases. **f** genitourinary system diseases. **g** musculoskeletal system diseases. **h** other diseases. COVID coronavirus disease, COPD chronic obstructive pulmonary disease, ESRD end-stage renal disease, No. number, AMSTAR 2 A Measurement Tool to Assess Systematic Reviews 2, CI confidence interval

The study also revealed that periodontal disease significantly elevates the risk of mortality from cerebrovascular diseases by 3.11 times, ischemic stroke by 2.88 times, Alzheimer’s disease and cognitive impairment by 1.67 times, and stroke by 1.32 times, highlighting its role as a significant risk factor for neurological disorders (Fig. 6a). No statistical significance was observed in the effect of periodontitis on dementia (*OR* = 1.59, 95% CI 0.92 − 2.76). However, the analysis indicated that periodontal disease was associated with an increased risk of dementia in patients with moderate or severe periodontitis (*OR* = 2.13, 95% CI 1.25 − 3.64). Periodontal disease also raises the risk of cognitive disorders (Fig. 6b). Additionally, periodontal disease was linked to a heightened risk of some respiratory diseases, such as pneumonia (*OR* = 3.21, 95% CI 2.00 − 5.17) and chronic obstructive pulmonary disease (COPD; *OR* = 1.20, 95% CI 1.09 − 1.32). However, the association did not reach statistical significance for pneumonia mortality, and acute exacerbation or mortality related to COPD (Fig. 6c). The study also found that periodontal disease increases the risk of pre-eclampsia by 1.43 times, premature rupture of the amniotic sac by 1.10 times, and gestational diabetes by 1.39 times (Fig. 6d). And the findings indicate that periodontal disease may contribute to a higher risk of preterm birth (*OR* = 1.57, 95% CI 1.39 − 1.77) and low weight at birth (*RR* = 2.19, 95% CI 1.82 − 2.64) among pregnant women (Fig. 6e). Moreover, periodontal disease was significantly correlated with an elevated risk of chronic kidney disease, exhibiting a *HR* of 1.60 (95% CI 1.44–1.79). In contrast, no significant increase in the risk of prostate inflammation was detected (*HR* = 1.32, 95% CI 0.87 − 1.77) (Fig. 6f). Furthermore, periodontal disease markedly heightened the risk of osteoporosis by 1.40 times, and rheumatoid arthritis by 1.69 times (Fig. 6g). It also demonstrated a significant association with age-related macular degeneration, increasing the risk by 1.35 times. Nevertheless, while several primary studies unveil an association between periodontal disease and the severity of diabetic retinopathy, the overall quality of evidence remains low, leaving this relationship still unclear. The study further revealed that periodontal disease substantially increased the risk of erectile dysfunction by 2.56 times and psoriasis by 2.87 times, as well as showing a notable correlation with obstructive sleep apnea, the risk increased by 2.17 times; however, this association was predominantly observed in cases involving mild to moderate periodontitis, without any significant link found in severe periodontitis cases. Besides, periodontal disease significantly elevated the risk of halitosis (*OR* = 4.05, 95% CI 1.76 − 9.30), exhibiting a robust association in both organoleptic testing and volatile sulfur compound reading. In addition, periodontal disease was notably linked to an increased risk of cardiac death (*RR* = 1.42, 95% CI 1.10 − 1.84) and all-cause mortality (*RR* = 1.31, 95% CI 1.07 − 1.61) (Fig. 6h).

## Discussion

This study encompassed 57 systematic reviews and meta-analyses aimed at investigating the relationship between periodontal disease and multiple systemic diseases. The results revealed a significant association between periodontal disease and systemic conditions, with an increased risk of head and neck cancer as well as circulatory system disorders such as coronary heart disease. Notably, approximately 71.93% of the studies were rated as having “Critically low” methodological quality. In conclusion, this study provides compelling evidence supporting the association between periodontal disease and multiple systemic diseases, underscoring the critical importance of effective management of periodontal health.

### Cancer

In recent years, the potential association between periodontal disease and cancer has gained significant attention [70]. Periodontal disease is a complex condition characterized by dynamic interactions among various pathogenic factors and host immune responses. The host immune response is modulated by an interplay of genetic and epigenetic influences, lifestyle choices, comorbidities, and dental health factors. Throughout the progression of different stages of periodontal disease, key mediators such as inflammatory cytokines and immune response play critical roles [71]. The chronic inflammatory state induced by periodontal disease, as an immune-related response, is considered a pivotal factor in this association. Prolonged inflammation may lead to systemic inflammation, continuous activation of the immune system, increased risk of DNA damage, and promotion of cancer development [72]. Studies have shown that individuals with periodontal disease exhibit a significantly elevated risk for cancers affecting the oral cavity, pancreas, esophagus, and colorectum [73–77]. This phenomenon may be attributed to the dissemination of periodontal pathogens and their metabolites to distant tissues via the bloodstream, resulting in localized inflammation [78, 79]. Among the 13 systematic reviews and meta-analyses incorporated in this study, the association between periodontal disease and 19 distinct types of cancer, including gastric adenocarcinoma, oral cancer, colorectal cancer, urogenital cancer, lung cancer, and pancreatic cancer, has been validated to varying extents. Although the study conducted by Xie et al. [65] did not find a significant association between periodontal disease and bladder cancer, the overall findings of the meta-analysis still support a positive correlation between periodontal disease and the risk of most cancer types, particularly head and neck cancer. It is closely linked to chronic inflammation and pathogen infection resulting from periodontal disease. Common pathogens such as *Porphyromonas gingivalis* may promote the development of oral cancers through multiple mechanisms [9], including dysregulation of the inflammatory microenvironment, inhibition of apoptosis, increased cellular proliferation, enhanced angiogenesis, promotion of epithelial-mesenchymal transition, and production of carcinogenic metabolites [80]. Additionally, the risk of pancreatic cancer is significantly increased in patients with periodontal disease, potentially related to systemic inflammation and immune system dysregulation [61, 68]. Periodontal pathogens and their toxins may be transmitted to the pancreas via the bloodstream, thereby altering the local tissue microenvironment and facilitating carcinogenesis. Wang et al. [34] indicated that while there is a moderate association between periodontal disease and the incidence of breast cancer, prostate cancer, and colorectal cancer, the relationship between the mortality rates of these cancers and periodontal disease remains nonsignificant. This discrepancy may stem from heterogeneity and statistical uncertainty present in the studies.

### Circulatory system diseases

The association between periodontal disease and circulatory system disorders has been widely investigated. The 9 included systematic reviews primarily focused on cardiovascular disease, including coronary heart disease and myocardial infarction, among other conditions. Most findings suggest a significant correlation between periodontal disease and these conditions, which is largely attributed to the systemic inflammatory response and vascular endothelial dysfunction induced by periodontal disease [81, 82]. As highlighted in the studies, the severity of periodontitis is associated with carotid intima-media thickness in young adults, and severe periodontitis and elevated leukocyte counts are independent risk factors for increased thickness, potentially linked to vascular endothelial injury [83, 84]. Additionally, periodontal pathogens and their metabolites may disseminate through the bloodstream, invading vascular endothelial cells, triggering localized vascular inflammatory responses, and promoting the development of arteriosclerosis and cardiovascular disease [85]. Furthermore, microorganisms can proliferate on atherosclerotic coronary plaques and worsen cardiovascular disease [86, 87]. However, the study by Chen et al. [13] involving patients with ESRD found no significant association between periodontitis and the risk of cardiovascular death in this population, suggesting that other factors may play a more critical role in determining cardiovascular death in this cohort.

### Digestive system diseases

The 6 studies included in this analysis explored the association between periodontal disease and various digestive system disorders, including liver disease, oral HPV infection, and inflammatory bowel disease. The findings indicated a correlation between periodontal disease and these digestive conditions, suggesting that periodontal disease may influence digestive system health through multiple mechanisms [88]. For instance, Larvin et al. [25] noted that while evidence is limited, periodontitis may increase the risk of liver disease, potentially due to the systemic inflammation induced by periodontal disease. Chronic inflammation can lead to abnormal immune responses in the liver, thereby promoting the development of liver disease [89]. Conversely, the study by Ali et al. [55] identified a significant association between periodontal disease and oral HPV infection; however, the relationship with high-risk oral HPV infection remains inconclusive. This may indicate that periodontal disease plays a complex and multifaceted role in oral and digestive tract infections [90, 91]. Given the intricacy of potential mechanisms linking periodontal disease to digestive system diseases [11], future research should employ more detailed molecular biology techniques and clinical studies to elucidate how periodontal disease affects digestive health through pathways such as inflammation and immune response, particularly focusing on its specific effects on various liver and gastrointestinal diseases.

### Endocrine, nutritional, or metabolic diseases

The association between periodontal disease and endocrine and metabolic disorders has garnered increasing attention. In this study, 7 systematic reviews and meta-analyses examined the relationship between periodontal disease and various metabolic conditions, such as diabetes, metabolic syndrome, and hyperlipidemia. Most findings indicate a significant correlation between periodontal disease and these metabolic disorders, particularly with diabetes and metabolic syndrome [92, 93]. Gobin et al. [59] provided reliable evidence for the association between periodontitis and metabolic syndrome, while Rosário-Dos-Santos et al. [21] further demonstrated a dose-response relationship showing that as the severity of the periodontal disease increases, so does the risk of developing metabolic syndrome. Periodontal disease may exacerbate metabolic syndrome by inducing systemic inflammation and insulin resistance [94, 95]. However, the study by Sayeed et al. [44] involving female populations found no significant association between periodontal disease and metabolic syndrome. Some studies have also suggested that gender may influence the prevalence and risk estimates of periodontal disease about other diseases, indicating that gender could play a moderating role in this relationship [23]. Overall, while numerous studies support the association between periodontal disease and endocrine and metabolic disorders, there exists some heterogeneity in findings across different investigations, potentially attributable to factors such as study population characteristics, research design methodologies, and criteria used for defining periodontal disease.

### Others

In addition to its association with cancer, circulatory, digestive, and metabolic diseases, periodontal disease is potentially linked to a variety of other systemic conditions. 5 studies have reported an association between periodontal disease and neurological disorders, including stroke, Alzheimer’s disease, and related outcomes [14, 37, 38, 50, 67]. In the context of mental health as well as behavioral and neurodevelopmental disorders, periodontal disease has been linked to mild cognitive impairment, cognitive decline, dementia, and anxiety [18, 23, 41, 45]. Although some studies have not identified a significant association between periodontal disease and dementia, the increased risk of dementia associated with moderate to severe periodontal disease suggests that periodontal disease may impact brain function through chronic inflammation and immune responses [23, 41]. Meta-analyses suggest that periodontal disease may increase the risk of COPD, pneumonia, and severe acute respiratory syndrome coronavirus 2 (SARS-CoV-2) infection [coronavirus disease 2019 (COVID-19)]. For instance, one study found that periodontal disease is associated with an elevated risk of pneumonia, while another indicated that it may heighten the risk of mortality related to COVID-19 [24, 51]. These findings imply that periodontal disease could exacerbate respiratory diseases by altering the respiratory microbiome and local immune response. Additionally, periodontal disease is linked to pregnancy-related complications, such as pre-eclampsia, premature rupture of the amniotic sac, and gestational diabetes, potentially due to the systemic inflammatory response and release of bioactive mediators triggered by periodontal inflammation [30]. Regarding urogenital diseases, periodontal disease has been related to chronic kidney disease and diabetic nephropathy; however, its relationship with prostate inflammation remains unclear [26, 50, 63]. There is also evidence connecting periodontal disease with osteoporosis, rheumatoid arthritis, and visual system disorders such as age-related macular degeneration [25, 52, 53]. Associations between periodontal disease and conditions related to sexual health such as erectile dysfunction, and skin diseases like psoriasis, have also been reported [33, 46]. Studies suggest that periodontal disease may increase the risk of these conditions by affecting systemic vascular function and immune responses [96, 97]. While these associations are supported by epidemiological studies, establishing causality necessitates further investigation through mechanistic research and clinical trials.

### Limitation and perspective

Despite analyzing a substantial number of systematic reviews and meta-analyses to explore the association between periodontal disease and systemic diseases, this study has several limitations. First, most primary studies included in systematic reviews and meta-analyses were cross-sectional or observational, making it challenging to establish causality. Clinical data were also not directly analyzed in this study. Second, this study did not specifically classify periodontal disease nor investigate the effects of various evolutionary stages such as gingivitis, mild to moderate periodontitis, and moderate to advanced periodontitis on systemic diseases. Third, most studies have inadequately controlled for confounding factors such as smoking habits, dietary patterns, and socioeconomic status, potentially compromising the accuracy of their findings. Additionally, heterogeneity among study populations and inconsistent diagnostic criteria for periodontal disease complicate the interpretation of results. Future research should prioritize: 1) investigating the causal relationship between periodontal disease and systemic diseases through well-designed longitudinal studies and randomized controlled trials; 2) evaluating the effects of different periodontal therapies on both prevention and treatment outcomes of systemic conditions; 3) exploring molecular and genetic mechanisms underlying the association between periodontal disease and systemic disorders to identify new diagnostic and therapeutic targets; and 4) enhancing public health efforts to prevent and control periodontal disease, particularly through early intervention in high-risk populations, thereby mitigating its potential adverse effects on overall health.

## Conclusions

Periodontal disease has been linked to a range of systemic conditions, including cancer, cardiovascular diseases, digestive disorders, endocrine and metabolic diseases, as well as neurological disorders. This condition not only shows a significant correlation with the onset of these diseases but may also influence their prognosis. However, the methodological quality of existing systematic reviews and meta-analyses is generally suboptimal, highlighting the need for improvement to generate high-quality evidence. Furthermore, establishing causality requires additional mechanistic research and high-quality randomized controlled trials.

## Supplementary Information

**Additional file 1**: **Table S1** Modified version of A Measurement Tool to Assess Systematic Reviews 2. **Table S2** Summary of basic information of included studies. **Table S3** Detailed evaluation of the methodological quality with modified version of A Measurement Tool to Assess Systematic Reviews 2

## Data Availability

The datasets generated during the current study will be available from the corresponding author upon reasonable request.

## Abbreviations

AMSTAR 2: A Measurement Tool to Assess Systematic Reviews 2
CI: Confidence interval
COPD: Chronic obstructive pulmonary disease
COVID-19: Coronavirus disease 2019
CVD: Cardiovascular disease
ESRD: End-stage renal disease
HPV: Human papillomavirus
HR: Hazard ratio
MeSH: Medical Subject Headings
MetS: Metabolic syndrome
OR: Odds ratio
OSA: Obstructive sleep apnea
RR: Relative risk
SARS-CoV-2: Severe acute respiratory syndrome coronavirus 2
T2DM: Type 2 diabetes mellitus
