# Supplementary material for: Association between periodontal disease and systemic diseases: a cross-sectional analysis of current evidence
Source: Mil Med Res. 2024 Dec 4;11:74. doi: 10.1186/s40779-024-00583-y (PMC11616297; doi:10.1186/s40779-024-00583-y)
Supplement: Supplementary file 1 — Additional file 1: Table S1 Modified version of A Measurement Tool to Assess Systematic Reviews 2. Table S2 Summary of basic information of included studies. Table S3 Detailed evaluation of the methodological quality with modified version of A Measurement Tool to Assess Systematic Reviews 2 [file 40779_2024_583_MOESM1_ESM.pdf]

Table S1 Modified version of A Measurement Tool to Assess Systematic Reviews 2

1. Did the research questions and inclusion criteria for the review include the key components of the focused theme?
For example, systematic reviews that include case control studies, cohort studies, cross-sectional studies of the association of certain exposure factors and relevant outcomes.

For Yes:

☐ population

☐ exposure factors

☐ comparator

☐ outcomes

☐ Yes
☐ No

2. Did the report of the review contain an explicit statement that the review methods were established prior to the conduct of the review and did the report justify any significant deviations from the protocol?

For Partial Yes:
The authors state that they had a written protocol or guide that included ALL the following:

☐ review question(s)

☐ a search strategy

☐ inclusion/exclusion criteria

☐ a risk of bias assessment

For Yes:
As for Partial Yes, plus the protocol should be registered and should also have specified:

☐ a meta-analysis/synthesis plan, if appropriate, and

☐ a plan for investigating causes of heterogeneity

☐ justification for any deviations from the protocol

☐ Yes
☐ Partial Yes
☐ No

3. Did the review authors explain their selection of the study designs for inclusion in the review?

For Yes, the review should satisfy the following:

☐ explanation for including only cross-sectional studies

☐ or explanation for including only case control studies

☐ or explanation for including only cohort studies

☐ or explanation for including two or more of cross-sectional studies, case control studies, cohort studies

☐ Yes
☐ No

4. Did the review authors use a comprehensive literature search strategy?

For Partial Yes (ALL the following):

☐ searched at least 2 databases (relevant to research question)
☐ provided key word and/or search strategy
☐ justified publication restrictions (eg, language)

For Yes, should also have (ALL the following):

☐ searched the reference lists / bibliographies of included studies
☐ searched trial/study registries
☐ included/consulted content experts in the field where relevant, searched for grey literature
☐ conducted search within 24 months of completion of the review

☐ Yes
☐ Partial Yes
☐ No

5. Did the review authors perform study selection in duplicate?

For Yes, either ONE of the following:

☐ at least 2 reviewers independently agreed on selection of eligible studies and achieved consensus on which studies to include
☐ OR 2 reviewers selected a sample of eligible studies and achieved good agreement (at least 80 percent), with the remainder selected by one reviewer

☐ Yes
☐ No

6. Did the review authors perform data extraction in duplicate?

For Yes, either ONE of the following:

☐ at least 2 reviewers achieved consensus on which data to extract from included studies
☐ OR 2 reviewers extracted data from a sample of eligible studies and achieved good agreement (at least 80 percent), with the remainder extracted by one reviewer

☐ Yes
☐ No

7. Did the review authors provide a list of excluded studies and justify the exclusions?

For Partial Yes:

☐ provided a list of all potentially relevant studies that were read in full-text form but excluded from the review

For Yes, must also have:

☐ justified the exclusion from the review of each potentially relevant study

☐ Yes
☐ Partial Yes
☐ No

8. Did the review authors describe the included studies in adequate detail?

For Partial Yes (ALL the following):

☐ described populations
☐ described exposure factors
☐ described comparators
☐ described outcomes
☐ described research designs

☐ Yes
☐ Partial Yes
☐ No

For Yes, should also have ALL the following:

☐ described population in detail

☐ described exposure factors in detail

☐ described comparators in detail

☐ described study's setting

9. Did the review authors use a satisfactory technique for assessing the risk of bias in individual studies that were included in the review?

Cohort studies

For Partial Yes, must have assessed:

☐ from confounding, and

☐ from selection bias

☐ Yes
☐ Partial Yes
☐ No

For Yes, must also have assessed:

☐ methods used to ascertain exposure and outcomes, and

☐ adequate follow up period for outcome of interest

☐ record linkage of independent blind assessment of outcome

☐ adequacy of follow up of cohorts (complete follow up/subjects lost to follow up unlikely to introduce bias)

Case control studies

For Partial Yes, must have assessed:

☐ from confounding, and

☐ from selection bias

☐ Yes
☐ Partial Yes
☐ No

For Yes, must also have assessed:

☐ methods used to ascertain exposures and outcomes, and

☐ same method of ascertainment exposures for both groups

☐ same non-response rate for both groups

Cross-sectional studies

For Partial Yes, must have assessed:

☐ from confounding, and

☐ from selection bias

☐ Yes
☐ Partial Yes
☐ No

For Yes, must also have assessed:

☐ methods used to ascertain exposures and outcomes, and

☐ appropriate statistical analysis used

10. Did the review authors report on the sources of funding for the studies included in the review?

For Yes:

☐ must have reported on the sources of funding for individual studies included in the review

Note: reporting that the reviewers looked for this information but it was not reported by study authors also qualifies

☐ Yes
☐ No

11. If meta-analysis was performed did the review authors use appropriate methods for statistical combination of results?

For Yes:

☐ the authors justified combining the data in a meta-analysis

☐ and they used an appropriate weighted technique to combine study results and adjusted for heterogeneity if present.

☐ and investigated the causes of any heterogeneity

☐ Yes
☐ No
☐ No meta-analysis conducted

12. If meta-analysis was performed, did the review authors assess the potential impact of risk of bias in individual studies on the results of the meta-analysis or other evidence synthesis?

For Yes:

☐ included only low risk of bias individual studies

☐ or, if the pooled estimate was based on individual studies at variable risk of bias, the authors performed analyses to investigate possible impact of risk of bias on summary estimates of effect

☐ Yes
☐ No
☐ No meta-analysis conducted

13. Did the review authors account for risk of bias in individual studies when interpreting/discussing the results of the review?

For Yes:

☐ included only low risk of bias individual studies

☐ or, if individual studies with moderate or high risk of bias, provided a discussion of the likely impact of risk of bias on the results

☐ Yes
☐ No

14. Did the review authors provide a satisfactory explanation for, and discussion of, any heterogeneity observed in the results of the review?

For Yes:

☐ there was no significant heterogeneity in the results

☐ or if heterogeneity was present the authors performed an investigation of sources of any heterogeneity in the results and discussed the impact of this on the results of the review

☐ Yes
☐ No

15. If they performed quantitative synthesis did the review authors carry out an adequate investigation of publication bias (small study bias) and discuss its likely impact on the results of the review?

For Yes:

☐ performed graphical or statistical tests for publication discussed likelihood and magnitude of impact of publication bias

☐ Yes
☐ No
☐ No meta-analysis conducted

16. Did the review authors report any potential sources of conflict of interest, including any funding they received for conducting the review?

For Yes:

☐ the authors reported no competing interests or

☐ the authors described their funding sources and how they managed potential conflicts of interest

☐ Yes
☐ No

Table S2 Summary of basic information of included studies

Study	Country	Journal	Study population	Number of included studies	Exposure (periodontal disease)	Outcome (systemic disease)	Conclusion
Chen et al. 2024 [1]	China	Med Oral Patol Oral Cir Bucal	End-stage renal disease (ESRD) patients	8	Periodontitis	Cardiovascular mortality, all-cause mortality	Periodontitis does not impact all-cause and cardiovascular mortality in ESRD patients
Dewan et al. 2024 [2]	India	Dent Med Probl	Adults	13	Periodontitis and gingivitis	Stroke (ischemic stroke, hemorrhagic stroke, transient ischemic attacks)	A significant association between stroke and periodontal disease
Ma et al. 2024 [3]	China	BMC Oral Health	Human	73	Periodontitis	Dyslipidemia	Periodontal disease is a risk factor for high triglyceride and low high-density lipoprotein levels
Aguiar et al. 2024 [4]	Brazil	Clinics	Human	9	Periodontal disease	Gastric adenocarcinoma	The presence of periodontal disease increased the risk of gastric adenocarcinoma
Li et al. 2024 [5]	China	Clin Oral Investig	Human	18	Periodontitis	Dental caries (crown caries, root caries)	A positive correlation between dental caries and periodontitis clinically
Lin et al. 2024 [6]	China	Dement Geriatr Cogn Disord	Adults	7	Periodontitis	Mild cognitive impairment	Patients with periodontitis are at a higher risk of developing mild cognitive impairment
Ma et al. 2024 [7]	China	Acta Odontol Scand	Human	16	Periodontitis	Oral cancer	Periodontitis may serve as a potential risk factor for oral cancer
Wang et al. 2024 [8]	China	Clin Oral Investig	Human	9	Periodontitis	Halitosis	A positive association was observed between halitosis and periodontitis
Rosário-Dos-Santos et al. 2023 [9]	Brazil	Oral Dis	Adults	14	Periodontitis	Metabolic syndrome	Moderate and severe levels of periodontitis are associated with metabolic syndrome
Leelaviwat et al. 2023 [10]	USA	J Arrhythm	Human	4	Periodontal disease	Atrial fibrillation or atrial flutter	Periodontal disease increases the risk of atrial fibrillation and atrial flutter

Study	Country	Journal	Study population	Number of included studies	Exposure (periodontal disease)	Outcome (systemic disease)	Conclusion
Larvin et al. 2023 [11] [a]	UK	Age Ageing	Human	39	Periodontal disease	Cognitive disorders (dementia and Alzheimer's disease, cognitive decline)	Periodontal disease demonstrated increased risks of cognitive disorders
Molina et al. 2023 [12]	Spain	J Clin Periodontol	Adults	75	Periodontitis	Respiratory diseases [asthma, severe acute respiratory syndrome coronavirus 2 (SARS CoV-2) infection]	A positive association between periodontitis and coronavirus disease 2019 (COVID-19) complications has been found, but not for asthma
Larvin et al. 2023 [13] [b]	UK	Community Dent Oral Epidemiol	Populations without predefined systemic disease	30	Periodontitis	Immune-mediated systemic conditions (osteoporosis, liver disease)	The risks of developing subsequent liver disease and osteoporosis are significantly increased, though the evidence for the associations is scarce
Ortíz de Urbina Comerón et al. 2023 [14]	Spain	J Clin Med	Men	7	Periodontal disease	Prostate inflammation	The incidence of periodontal disease does not increase the risk of the incidence of prostate inflammation
Yang et al. 2023 [15]	China	BMJ Open	Adults	22	Periodontal disease	Chronic obstructive pulmonary disease (COPD), COPD-related acute exacerbation or mortality	Periodontal disease confers no risk for COPD and COPD-related events when strictly adjusted by smoking
Guo et al. 2023 [16]	China	PLoS One	Adults free of cardiovascular disease (CVD) at initially	39	Moderate or severe periodontal disease	Cardiovascular outcome (coronary heart disease, stroke, cardiac death), all-cause mortality	Periodontal disease were associated with an increased risk of coronary heart disease, stroke, cardiac death, and all-cause mortality
Xu et al. 2023 [17]	China	Community Dent Health	Human	7	Periodontitis	Non-alcoholic fatty liver disease	Current evidence fails to demonstrate a link between periodontitis and non-alcoholic fatty liver disease

Study	Country	Journal	Study population	Number of included studies	Exposure (periodontal disease)	Outcome (systemic disease)	Conclusion
Karimi et al. 2023 [18]	Iran	Health Sci Rep	Pregnant women	67	Periodontal disease	Adverse maternal or neonatal outcomes (pre-eclampsia, premature rupture of the amniotic sac, gestational diabetes, low weight at birth)	Periodontal disease may contribute to a higher risk of poor maternal and newborn outcomes in pregnant women
Khodadadi et al. 2022 [19]	Iran	J Clin Exp Dent	Adults	10	Periodontitis	Obstructive sleep apnea (OSA)	Periodontitis has a direct association with OSA. Periodontitis has been shown to be associated with mild-to-moderate OSA, but not with severe OSA
Zhang et al. 2022 [20]	China	Front Pediatr	Pregnant women	24	Periodontal disease	Adverse neonatal outcome (preterm birth, small for gestational age)	Pregnant women with periodontal disease have a significantly higher risk of preterm birth. No relationship between periodontal disease and small for gestational age
Zhang et al. 2022 [21]	China	Oral Dis	Adults	13	Periodontitis	Psoriasis	A positive association between periodontitis and psoriasis
Wang et al. 2022 [22]	China	PeerJ	Human	27	Periodontal disease	Cancer (breast, prostate, colon and rectum)	A modest association between periodontal disease and breast, prostate, colon and rectum cancer incidence. Periodontal disease may increase the risk of breast, prostate, colon and rectum cancer mortality, the overall estimate was not significant
Li et al. 2022 [23]	China	Front Oncol	Adults	11	Periodontal disease	Urogenital cancer	Periodontal disease is a potential risk factor for urogenital cancer
Kesharani et al. 2022 [24]	India	Contemp Clin Dent	Human	12	Periodontitis	Lung cancer	Periodontal disease is a potential risk factor for the development of lung cancer

Study	Country	Journal	Study population	Number of included studies	Exposure (periodontal disease)	Outcome (systemic disease)	Conclusion
Kaliamoorthy et al. 2022 [25]	India	Med Pharm Rep	Adults	5	Periodontitis	Alzheimer's disease (Alzheimer's disease or cognitive impairment)	A significant association between periodontitis and Alzheimer's disease
Romandini et al. 2021 [26]	Spain	J Dent Res	Human	57	Periodontitis	Mortality (cancer, coronary heart disease, cerebrovascular diseases, pneumonia)	Periodontitis is associated with an increased risk of all-cause and cause-specific mortality, but not for pneumonia
Larvin et al. 2021 [27]	UK	Clin Exp Dent Res	Population free from predefined systemic disease at baseline	32	Periodontal disease	CVD	Modest but consistently increased risk of CVD in periodontal disease populations
Qin et al. 2021 [28]	China	AM J EMERG MED	Not defined	10	Periodontal disease	Myocardial infarction	Periodontal disease is modestly associated with myocardial infarction, especially in women
Guo et al. 2021 [29]	China	Int J Environ Res Public Health	Human	20	Periodontitis	Dementia	No statistical significance in the effect of periodontitis on dementia. Moderate or severe periodontitis was a risk factor for dementia
Alvarenga et al. 2021 [30]	Brazil	Front Public Health	Humans with diabetes mellitus	5	Periodontitis	Diabetic retinopathy	An association between diabetic retinopathy and periodontitis, however, the quality of the body of evidence was low
Stöhr et al. 2021 [31]	Germany	Sci Rep	Humans, except pregnant women, children and adolescents	15	Periodontal disease	Diabetes mellitus	A positive bidirectional association between periodontal disease and diabetes mellitus
Sayeed et al. 2021 [32]	Saudi Arabia	J INT SOC PREV COMMU	Female	37	Periodontal disease	Metabolic syndrome	Lack of convincing proof of a link between metabolic syndrome and periodontal disease in females

Study	Country	Journal	Study population	Number of included studies	Exposure (periodontal disease)	Outcome (systemic disease)	Conclusion
Aragão et al. 2021 [33]	Brazil	Front Psychiatry	Adults	11	Periodontitis	Anxiety	Positive association between periodontitis and anxiety, although with very low certainty of evidence
Farook et al. 2021 [34]	Saudi Arabia	Am J Mens Health	Adult males	6	Periodontitis	Erectile dysfunction	A significant association exists between the periodontal disease and erectile dysfunction
Wu et al. 2020 [35]	China	Med Oral Patol Oral Cir Bucal	Patients of any age	6	Periodontitis	Hematopoietic and lymphatic cancers	Periodontal disease might be considered as a risk factor for hematopoietic and lymphatic cancers
Zhang et al. 2020 [36]	China	J Clin Periodontol	Human	10	Periodontitis	Gastrointestinal cancers	Periodontitis may be a risk factor for gastrointestinal cancers
Gopinath et al. 2020 [37]	Malaysia	Cancers	Human	21	Periodontal disease	Head and neck cancer	Periodontal disease is an independent risk factor for head and neck cancer
Nguyen et al. 2020 [38]	Australia	Diabetes Res Clin Pract	Participants with diabetes mellitus	14	Periodontal disease	Complications of diabetes mellitus (neuropathy, nephropathy, cardiovascular complications and death)	A link between poor oral health and diabetes mellitus and its complications
Gomes-Filho et al. 2020 [39]	Brazil	Oral Dis	Adults	13	Periodontitis	Respiratory diseases (pneumonia)	An association between periodontitis and pneumonia
Lv et al. 2020 [40]	China	Biomed Res Int	Human	5	Periodontal disease	Age-related macular degeneration	Periodontal disease patients have a higher risk of age-related macular degeneration
Qiao et al. 2020 [41]	China	Joint Bone Spine	Human	13	Periodontitis	Rheumatoid arthritis	An increased risk of rheumatoid arthritis in patients with periodontitis

Study	Country	Journal	Study population	Number of included studies	Exposure (periodontal disease)	Outcome (systemic disease)	Conclusion
Hussain et al. 2020 [42]	UK	Semin Arthritis Rheum	Adults with rheumatoid arthritis	6	Periodontitis	Rheumatoid arthritis (clinical activity)	Periodontal disease is associated with worse rheumatoid arthritis clinical activity
Ali et al. 2020 [43]	Australia	J Public Health	Human	13	Periodontitis	Oral human papillomavirus (HPV) infection	A positive association between periodontitis and oral HPV infection. Evidence for an association between periodontitis and high-risk oral HPV infection is inconclusive
Xu et al. 2020 [44]	China	Clin Exp Pharmacol Physiol	Human	29	Periodontitis	Hyperlipidaemia	Periodontal disease is indeed associated with hyperlipidemia in humans
Aguilera et al. 2020 [45]	UK	Cardiovasc Res	Individuals (≥16 years)	81	Periodontitis	Hypertension	Periodontitis could be associated with increased risk of hypertension in a linear fashion
Machado et al. 2020 [46]	Portugal	J Clin Med	Female patients	12	Periodontitis	Polycystic ovary syndrome	A higher risk of individuals with polycystic ovary syndrome being diagnosed with periodontal disease
Gobin et al. 2020 [47]	China	Front Endocrinol	Adults	43	Periodontal disease	Metabolic syndrome	Compelling evidence for the association between periodontitis and metabolic syndrome
Lorenzo-Pouso et al. 2020 [48]	Spain	Acta Odontol Scand	Patients	9	Periodontal disease	Inflammatory bowel disease	Clear evidence for an association between periodontal disease and inflammatory bowel disease
Ma et al. 2020 [49]	China	Int J Med Sci	Human	10	Periodontitis	Cancer (esophageal cancer, hematological malignancy, melanoma of skin)	A potential link between periodontitis and esophageal cancer, hematological malignancy, and melanoma of the skin
Wu et al. 2020 [50]	China	BMC Oral Health	Human	53	Periodontitis	Type 2 diabetes mellitus (T2DM)	An evident bidirectional relationship between T2DM and periodontitis

Study	Country	Journal	Study population	Number of included studies	Exposure (periodontal disease)	Outcome (systemic disease)	Conclusion
Kapellas et al. 2019 [51]	Australia	Nephrology	Adults	17	Periodontal disease	Chronic kidney disease	Moderate evidence for a positive association between periodontitis and chronic kidney disease exists
Ferreira et al. 2018 [52]	Brazil	J Dent	Human	19	Periodontitis	Peri-implantitis	Diagnosis or history of periodontitis was associated with the occurrence of peri-implantitis
Xie et al. 2018 [53]	China	Front Physiol	Human	5	Periodontal disease	Bladder cancer	Patients with periodontal disease may not be at an increased risk of developing bladder cancer
Corbella et al. 2018 [54]	Italy	PLoS One	Human	10	Periodontitis	Cancer (digestive tract cancer, corpus uteri cancer, esophagus or oropharyngeal cancer, non-Hodgkin lymphoma)	A low but statistically significant association between periodontitis and different types of cancer
Leira et al. 2017 [55]	Spain	Eur J Epidemiol	Adults	8	Periodontitis	Ischemic stroke	An association between periodontitis and ischemic stroke
Maisonneuve et al. 2017 [56]	USA	Ann Oncol	Human	8	Periodontitis	Pancreatic cancer	An association between periodontal disease and subsequent tooth loss with pancreatic cancer
Zeng et al. 2016 [57]	China	Int J Cardiol	Human	15	Periodontal disease	Carotid atherosclerosis	Periodontal disease was associated with carotid atherosclerosis

[a,b] Different studies published by the same author in the same year. ESRD end-stage renal disease, SARS-CoV-2 severe acute respiratory syndrome coronavirus 2, COVID-19 coronavirus disease 2019, COPD chronic obstructive pulmonary disease, CVD cardiovascular disease, OSA obstructive sleep apnea, HPV human papillomavirus, T2DM type 2 diabetes mellitus

Table S3 Detailed evaluation of the methodological quality with modified version of A Measurement Tool to Assess Systematic Reviews 2

Study	Q1	Q2	Q3	Q4	Q5	Q6	Q7	Q8	Q9	Q10	Q11	Q12	Q13	Q14	Q15	Q16	All
Chen et al. 2024 [1]	Y	PY	N	PY	Y	Y	N	Y	Y	N	Y	N	N	N	Y	Y	Critically low
Dewan et al. 2024 [2]	Y	PY	N	PY	N	Y	N	Y	PY	N	N	N	Y	Y	N	Y	Critically low
Ma et al. 2024 [3]	Y	N	N	PY	Y	N	N	Y	Y	N	Y	N	N	Y	Y	Y	Critically low
Aguiar et al. 2024 [4]	Y	PY	N	PY	Y	Y	N	Y	Y	N	N	N	Y	N	Y	Y	Critically low
Li et al. 2024 [5]	Y	N	N	PY	N	Y	N	Y	Y	N	Y	N	N	N	Y	Y	Critically low
Lin et al. 2024 [6]	Y	PY	N	PY	Y	N	N	Y	Y	N	N	N	N	Y	N	Y	Critically low
Ma et al. 2024 [7]	Y	PY	N	PY	Y	N	N	Y	Y	N	Y	N	N	Y	Y	Y	Critically low
Wang et al. 2024 [8]	Y	PY	N	PY	Y	N	N	Y	PY	N	N	N	Y	Y	N	Y	Critically low
Rosário-Dos-Santos et al. 2023 [9]	Y	PY	N	PY	Y	Y	Y	Y	PY	N	Y	N	Y	Y	Y	Y	Moderate
Leelaviwat et al. 2023 [10]	Y	N	N	PY	N	N	N	Y	N	N	N	N	N	N	N	Y	Critically low
Larvin et al. 2023 [11] [a]	Y	PY	N	PY	N	N	N	PY	PY	N	Y	N	Y	Y	Y	Y	Low
Molina et al. 2023 [12]	Y	PY	N	PY	Y	Y	Y	Y	Y	Y	N	N	Y	Y	Y	Y	Low
Larvin et al. 2023 [13] [b]	Y	PY	N	PY	Y	Y	Y	PY	PY	N	Y	N	Y	Y	Y	Y	Moderate
Ortíz de Urbina Comerón et al. 2023 [14]	Y	N	N	PY	Y	Y	N	PY	Y	N	N	N	Y	N	Y	Y	Critically low
Yang et al. 2023 [15]	Y	N	N	PY	Y	Y	N	PY	PY	N	Y	N	Y	Y	Y	Y	Critically low
Guo et al. 2023 [16]	Y	N	N	PY	Y	Y	N	PY	Y	N	Y	Y	Y	Y	Y	Y	Critically low
Xu et al. 2023 [17]	Y	PY	N	PY	Y	Y	N	PY	PY	N	Y	N	N	Y	Y	Y	Critically low
Karimi et al. 2023 [18]	Y	N	N	PY	Y	Y	N	Y	Y	N	N	N	Y	N	Y	Y	Critically low
Khodadadi et al. 2022 [19]	Y	N	N	PY	Y	Y	N	PY	Y	N	Y	N	N	Y	Y	Y	Critically low

**Study**	**Q1**	**Q2**	**Q3**	**Q4**	**Q5**	**Q6**	**Q7**	**Q8**	**Q9**	**Q10**	**Q11**	**Q12**	**Q13**	**Q14**	**Q15**	**Q16**	**All**
Zhang et al. 2022 [20]	Y	N	N	PY	Y	Y	N	Y	Y	N	Y	N	N	Y	Y	Y	Critically low
Zhang et al. 2022 [21]	Y	PY	N	PY	Y	Y	N	Y	Y	N	Y	N	Y	Y	N	Y	Critically low
Wang et al. 2022 [22]	Y	PY	N	PY	Y	Y	N	Y	Y	N	Y	N	Y	Y	Y	Y	Low
Li et al. 2022 [23]	Y	PY	N	PY	Y	N	Y	Y	Y	N	Y	Y	Y	Y	Y	Y	Moderate
Kesharani et al. 2022 [24]	Y	PY	N	PY	N	N	N	Y	Y	N	Y	Y	Y	Y	N	Y	Critically low
Kaliamoorthy et al. 2022 [25]	Y	PY	N	PY	Y	Y	N	PY	PY	N	N	N	N	N	N	N	Critically low
Romandini et al. 2021 [26]	Y	PY	N	PY	Y	Y	Y	Y	Y	N	Y	Y	Y	Y	Y	Y	Moderate
Larvin et al. 2021 [27]	Y	PY	N	PY	Y	Y	N	PY	PY	N	Y	N	Y	Y	Y	Y	Low
Qin et al. 2021 [28]	Y	N	N	PY	N	N	N	Y	Y	N	Y	Y	Y	Y	Y	Y	Critically low
Guo et al. 2021 [29]	Y	N	N	PY	Y	Y	Y	Y	PY	N	Y	N	N	Y	Y	Y	Critically low
Alvarenga et al. 2021 [30]	Y	PY	N	PY	Y	N	N	Y	Y	N	NA	NA	N	N	NA	Y	Critically low
Stöhr et al. 2021 [31]	Y	PY	N	PY	Y	Y	Y	Y	Y	N	Y	Y	Y	Y	Y	Y	Moderate
Sayeed et al. 2021 [32]	Y	N	N	PY	N	N	N	PY	PY	N	Y	N	Y	Y	N	Y	Critically low
Aragão et al. 2021 [33]	Y	PY	N	PY	Y	Y	Y	Y	Y	N	NA	NA	Y	Y	NA	Y	Moderate
Farook et al. 2021 [34]	Y	PY	N	PY	Y	N	N	Y	PY	N	Y	N	Y	Y	N	Y	Critically low
Wu et al. 2020 [35]	Y	N	N	PY	N	Y	Y	Y	Y	N	Y	N	Y	Y	Y	Y	Low
Zhang et al. 2020 [36]	Y	N	N	PY	Y	Y	N	Y	Y	N	Y	Y	Y	Y	Y	Y	Critically low
Gopinath et al. 2020 [37]	Y	PY	N	PY	Y	Y	N	PY	Y	N	Y	N	Y	Y	Y	Y	Low
Nguyen et al. 2020 [38]	Y	N	N	PY	Y	N	N	Y	PY	N	NA	NA	N	N	NA	Y	Critically low
Gomes-Filho et al. 2020 [39]	Y	PY	N	PY	Y	Y	N	Y	PY	N	N	Y	Y	Y	Y	Y	Critically low

Study	Q1	Q2	Q3	Q4	Q5	Q6	Q7	Q8	Q9	Q10	Q11	Q12	Q13	Q14	Q15	Q16	All
Lv et al. 2020 [40]	Y	N	N	PY	Y	Y	N	Y	Y	N	Y	Y	Y	Y	Y	Y	Critically low
Qiao et al. 2020 [41]	Y	N	N	PY	Y	Y	N	Y	PY	N	Y	N	N	Y	Y	Y	Critically low
Hussain et al. 2020 [42]	Y	PY	N	PY	Y	Y	Y	PY	Y	N	N	Y	Y	Y	N	Y	Critically low
Ali et al. 2020 [43]	Y	PY	N	PY	Y	N	Y	Y	PY	N	Y	N	Y	Y	N	N	Low
Xu et al. 2020 [44]	Y	N	N	PY	N	Y	N	PY	Y	N	Y	N	N	Y	Y	Y	Critically low
Aguilera et al. 2020 [45]	Y	PY	N	PY	Y	N	N	Y	PY	N	Y	Y	N	Y	Y	Y	Critically low
Machado et al. 2020 [46]	Y	N	N	PY	Y	Y	Y	PY	Y	Y	Y	Y	Y	Y	N	Y	Critically low
Gobin et al. 2020 [47]	Y	N	N	PY	N	Y	N	Y	PY	N	Y	N	N	Y	Y	Y	Critically low
Lorenzo-Pouso et al. 2020 [48]	Y	N	N	PY	Y	Y	N	Y	Y	N	Y	N	N	Y	Y	Y	Critically low
Ma et al. 2020 [49]	Y	N	N	PY	Y	N	N	PY	Y	N	Y	Y	Y	Y	N	Y	Critically low
Wu et al. 2020 [50]	Y	PY	N	PY	Y	Y	N	PY	Y	N	Y	Y	Y	Y	Y	Y	Low
Kapellas et al. 2019 [51]	Y	PY	N	PY	Y	N	N	Y	Y	N	Y	Y	N	Y	Y	Y	Critically low
Ferreira et al. 2018 [52]	Y	PY	N	PY	Y	N	N	Y	Y	N	Y	Y	Y	Y	Y	Y	Low
Xie et al. 2018 [53]	Y	N	N	PY	N	Y	Y	PY	N	N	Y	N	N	Y	N	Y	Critically low
Corbella et al. 2018 [54]	Y	PY	N	PY	Y	Y	Y	Y	PY	N	Y	Y	Y	Y	N	Y	Low
Leira et al. 2017 [55]	Y	PY	N	PY	Y	Y	N	Y	N	N	Y	N	N	Y	N	Y	Critically low
Maisonneuve et al. 2017 [56]	Y	N	N	N	Y	Y	N	Y	N	N	Y	N	N	Y	Y	Y	Critically low
Zeng et al. 2016 [57]	Y	N	N	PY	Y	Y	N	Y	N	N	Y	N	N	Y	Y	Y	Critically low

Q1 − Q16 16 evaluation items of modified version of A Measurement Tool to Assess Systematic Reviews 2, [a,b] Different studies published by the same author in the same year, Y Yes, N No, PY Partial Yes, NA No meta-analysis conducted

1. Chen H, Li J. Association between periodontitis and its treatment on mortality rates of end-stage renal disease: a systematic review and meta-analysis. Med Oral Patol Oral Cir Bucal. 2024;29(3):e334-42.
2. Dewan M, Pandit AK, Goyal L. Association of periodontitis and gingivitis with stroke: a systematic review and meta-analysis. Dent Med Probl. 2024;61(3):407-15.
3. Ma W, Zou Z, Yang L, Lin D, Guo J, Shan Z, et al. Exploring the bi-directional relationship between periodontitis and dyslipidemia: a comprehensive systematic review and meta-analysis. BMC Oral Health. 2024;24(1):508.
4. Aguiar FJN, Menezes FDS, Fagundes MA, Fernandes GA, Alves FA, Filho JG, et al. Gastric adenocarcinoma and periodontal disease: a systematic review and meta-analysis. Clinics (Sao Paulo). 2024;79:100321.
5. Li Y, Xiang Y, Ren H, Zhang C, Hu Z, Leng W, et al. Association between periodontitis and dental caries: a systematic review and meta-analysis. Clin Oral Investig. 2024;28(6):306.
6. Lin J, Pathak JL, Shen Y, Mashrah MA, Zhong X, Chen J, et al. Association between periodontitis and mild cognitive impairment: a systematic review and meta-analysis. Dement Geriatr Cogn Disord. 2024;53(1):37-46.
7. Ma Y, Tuerxun N, Maimaitili G. Periodontitis and the risk of oral cancer: a meta-analysis of case-control studies. Acta Odontol Scand. 2024;83:40478.
8. Wang N, Li C, Zhang J, Wang L, Yang J. The association between halitosis and periodontitis: a systematic review and meta-analysis. Clin Oral Investig. 2024;28(6):341.
9. Rosário-Dos-Santos HL, Miranda SS, Gomes-Filho IS, Cruz SSD, Figueiredo A, Souza ES, et al. Periodontitis severity relationship with metabolic syndrome: a systematic review with meta-analysis. Oral Dis. 2023;29(7):2512-20.
10. Leelaviwat N, Kewcharoen J, Poomprakobsri K, Trongtorsak A, Del Rio-Pertuz G, Abdelnabi M, et al. Periodontal disease and risk of atrial fibrillation or atrial flutter: a systematic review and meta-analysis. J Arrhythm. 2023;39(6):992-6.
11. Larvin H, Gao C, Kang J, Aggarwal VR, Pavitt S, Wu J. The impact of study factors in the association of periodontal disease and cognitive disorders: systematic review and meta-analysis. Age Ageing. 2023;52(2):afad015.
12. Molina A, Huck O, Herrera D, Montero E. The association between respiratory diseases and periodontitis: a systematic review and meta-analysis. J Clin Periodontol. 2023;50(6):842-87.
13. Larvin H, Kang J, Aggarwal VR, Pavitt S, Wu J. Periodontitis and risk of immune-mediated systemic conditions: a systematic review and meta-analysis. Community Dent Oral Epidemiol. 2023;51(5):705-17.

14. Ortíz de Urbina Comerón P, Zubizarreta-Macho Á, Lobo Galindo AB, Montiel-Company JM, Lorenzo-Gomez MF, Flores Fraile J. Relationship between prostate inflammation and periodontal disease-a systematic review and meta-analysis. J Clin Med. 2023;12(18):6070.

15. Yang M, Peng R, Li X, Peng J, Liu L, Chen L. Association between chronic obstructive pulmonary disease and periodontal disease: a systematic review and meta-analysis. BMJ Open. 2023;13(6):e067432.

16. Guo X, Li X, Liao C, Feng X, He T. Periodontal disease and subsequent risk of cardiovascular outcome and all-cause mortality: a meta-analysis of prospective studies. PLoS One. 2023;18(9):e0290545.

17. Xu F, Tang J. Is there an association between periodontitis and non-alcoholic fatty liver disease? A systematic review and meta-analysis. Community Dent Health. 2023;40(1):47-52.

18. Karimi N, Samiee N, Moradi Y. The association between periodontal disease and risk of adverse maternal or neonatal outcomes: a systematic review and meta-analysis of analytical observational studies. Health Sci Rep. 2023;6(10):e1630.

19. Khodadadi N, Khodadadi M, Zamani M. Is periodontitis associated with obstructive sleep apnea? A systematic review and meta-analysis. J Clin Exp Dent. 2022;14(4):e359-65.

20. Zhang Y, Feng W, Li J, Cui L, Chen ZJ. Periodontal disease and adverse neonatal outcomes: a systematic review and meta-analysis. Front Pediatr. 2022;10:799740.

21. Zhang X, Gu H, Xie S, Su Y. Periodontitis in patients with psoriasis: a systematic review and meta-analysis. Oral Dis. 2022;28(1):33-43.

22. Wang K, Zhang Z, Wang Z. Assessment of the association between periodontal disease and total cancer incidence and mortality: a meta-analysis. PeerJ. 2022;10:e14320.

23. Li W, Wang S, He Y, Zhang Y, Lin S, Cen D, et al. Is periodontal disease a risk indicator for urogenital cancer? A systematic review and meta-analysis of cohort studies. Front Oncol. 2022;12:697399.

24. Kesharani P, Kansara P, Kansara T, Kini A, Bhat R, Shetty P, et al. Is periodontitis a risk factor for lung cancer? A meta-analysis and detailed review of mechanisms of association. Contemp Clin Dent. 2022;13(4):297-306.

25. Kaliamoorthy S, Nagarajan M, Sethuraman V, Jayavel K, Lakshmanan V, Palla S. Association of Alzheimer's disease and periodontitis - a systematic review and meta-analysis of evidence from observational studies. Med Pharm Rep. 2022;95(2):144-51.

26. Romandini M, Baima G, Antonoglou G, Bueno J, Figuero E, Sanz M. Periodontitis, edentulism, and risk of mortality: a systematic review with meta-analyses. J Dent Res. 2021;100(1):37-49.

27. Larvin H, Kang J, Aggarwal VR, Pavitt S, Wu J. Risk of incident cardiovascular disease in people with periodontal disease: a systematic review and meta-analysis. Clin Exp Dent Res. 2021;7(1):109-22.

28. Qin X, Zhao Y, Guo Y. Periodontal disease and myocardial infarction risk: a meta-analysis of cohort studies. Am J Emerg Med. 2021;48:103-9.

29. Guo H, Chang S, Pi X, Hua F, Jiang H, Liu C, et al. The effect of periodontitis on dementia and cognitive impairment: a meta-analysis. Int J Environ Res Public Health. 2021;18(13):6823.

30. Alvarenga MOP, Miranda GHN, Ferreira RO, Saito MT, Fagundes NCF, Maia LC, et al. Association between diabetic retinopathy and periodontitis-a systematic review. Front Public Health. 2021;8:550614.

31. Stöhr J, Barbaresko J, Neuenschwander M, Schlesinger S. Bidirectional association between periodontal disease and diabetes mellitus: a systematic review and meta-analysis of cohort studies. Sci Rep. 2021;11(1):13686.

32. Sayeed G, Varghese SS. Association between periodontitis and metabolic syndrome in females: a systematic review and meta-analysis. J Int Soc Prev Community Dent. 2021;11(6):609-25.

33. Aragão WAB, Souza-Monteiro D, Frazão DR, Ne YGS, Ferreira RO, Rivera LFS, et al. Is there any association between chronic periodontitis and anxiety in adults? A systematic review. Front Psychiatry. 2021;12:710606.

34. Farook F, Al Meshrafi A, Mohamed Nizam N, Al Shammari A. The association between periodontitis and erectile dysfunction: a systematic review and meta-analysis. Am J Mens Health. 2021;15(3):15579883211007277.

35. Wu Y, Shi X, Li Y, Shi X, Gu Y, Qian Q, et al. Hematopoietic and lymphatic cancers in patients with periodontitis: a systematic review and meta-analysis. Med Oral Patol Oral Cir Bucal. 2020;25(1):e21-8.

36. Zhang Y, Sun C, Song EJ, Liang M, Shi T, Min M, et al. Is periodontitis a risk indicator for gastrointestinal cancers? A meta-analysis of cohort studies. J Clin Periodontol. 2020;47(2):134-47.

37. Gopinath D, Kunnath Menon R, Veettil SK, George Botelho M, Johnson NW. Periodontal diseases as putative risk factors for head and neck cancer: systematic review and meta-analysis. Cancers (Basel). 2020;12(7):1893.

38. Nguyen ATM, Akhter R, Garde S, Scott C, Twigg SM, Colagiuri S, et al. The association of periodontal disease with the complications of diabetes mellitus. A systematic review. Diabetes Res Clin Pract. 2020;165:108244.

39. Gomes-Filho IS, Cruz SSD, Trindade SC, Passos-Soares JS, Carvalho-Filho PC, Figueiredo A, et al. Periodontitis and respiratory diseases: a systematic review with meta-analysis. Oral Dis. 2020;26(2):439-46.

40. Lv X, Li W, Fang Z, Xue X, Pan C. Periodontal disease and age-related macular degeneration: a meta-analysis of 112,240 participants. Biomed Res Int. 2020;2020:4753645.

41. Qiao Y, Wang Z, Li Y, Han Y, Zhou Y, Cao X. Rheumatoid arthritis risk in periodontitis patients: a systematic review and meta-analysis. Joint Bone Spine. 2020;87(6):556-64.

42. Hussain SB, Botelho J, Machado V, Zehra SA, Mendes JJ, Ciurtin C, et al. Is there a bidirectional association between rheumatoid arthritis and periodontitis? A systematic review and meta-analysis. Semin Arthritis Rheum. 2020;50(3):414-22.

43. Ali A, Lassi ZS, Kapellas K, Jamieson L, Rumbold AR. A systematic review and meta-analysis of the association between periodontitis and oral high-risk human papillomavirus infection. J Public Health (Oxf). 2020;43(4):e610-9.

44. Xu J, Duan X. Association between periodontitis and hyperlipidaemia: a systematic review and meta-analysis. Clin Exp Pharmacol Physiol. 2020;47(11):1861-73.

45. Muñoz Aguilera E, Suvan J, Buti J, Czesnikiewicz-Guzik M, Barbosa Ribeiro A, Orlandi M, et al. Periodontitis is associated with hypertension: a systematic review and meta-analysis. Cardiovasc Res. 2020;116(1):28-39.

46. Machado V, Escalda C, Proença L, Mendes JJ, Botelho J. Is there a bidirectional association between polycystic ovarian syndrome and periodontitis? A systematic review and meta-analysis. J Clin Med. 2020;9(6):1961.

47. Gobin R, Tian D, Liu Q, Wang J. Periodontal diseases and the risk of metabolic syndrome: an updated systematic review and meta-analysis. Front Endocrinol (Lausanne). 2020;11:336.

48. Lorenzo-Pouso AI, Castelo-Baz P, Rodriguez-Zorrilla S, Pérez-Sayáns M, Vega P. Association between periodontal disease and inflammatory bowel disease: a systematic review and meta-analysis. Acta Odontol Scand. 2020;79(5):344-53.

49. Ma H, Zheng J, Li X. Potential risk of certain cancers among patients with periodontitis: a supplementary meta-analysis of a large-scale population. Int J Med Sci. 2020;17(16):2531-43.

50. Wu CZ, Yuan YH, Liu HH, Li SS, Zhang BW, Chen W, et al. Epidemiologic relationship between periodontitis and type 2 diabetes mellitus. BMC Oral Health. 2020;20(1):204.

51. Kapellas K, Singh A, Bertotti M, Nascimento GG, Jamieson LM, Perio CKDc. Periodontal and chronic kidney disease association: a systematic review and meta-analysis. Nephrology (Carlton). 2019;24(2):202-12.

52. Ferreira SD, Martins CC, Amaral SA, Vieira TR, Albuquerque BN, Cota LOM, et al. Periodontitis as a risk factor for peri-implantitis: systematic review and meta-analysis of observational studies. J Dent. 2018;79:1-10.

53. Xie WZ, Jin YH, Leng WD, Wang XH, Zeng XT, investigators B. Periodontal disease and risk of bladder cancer: a meta-analysis of 298476 participants. Front Physiol. 2018;9:979.

54. Corbella S, Veronesi P, Galimberti V, Weinstein R, Del Fabbro M, Francetti L. Is periodontitis a risk indicator for cancer? A meta-analysis. PLoS One. 2018;13(4):e0195683.

55. Leira Y, Seoane J, Blanco M, Rodriguez-Yanez M, Takkouche B, Blanco J, et al. Association between periodontitis and ischemic stroke: a systematic review and meta-analysis. Eur J Epidemiol. 2017;32(1):43-53.

56. Maisonneuve P, Amar S, Lowenfels AB. Periodontal disease, edentulism, and pancreatic cancer: a meta-analysis. Ann Oncol. 2017;28(5):985-95.

57. Zeng XT, Leng WD, Lam YY, Yan BP, Wei XM, Weng H, et al. Periodontal disease and carotid atherosclerosis: a meta-analysis of 17,330 participants. Int J Cardiol. 2016;203:1044-51.
